# 5-HT_1A_ Agonist Properties Contribute to a Robust Response to Vilazodone in the Novelty Suppressed Feeding Paradigm

**DOI:** 10.1093/ijnp/pyw057

**Published:** 2016-06-28

**Authors:** Alvaro L. Garcia-Garcia, Míriam Navarro-Sobrino, Gila Pilosof, Pradeep Banerjee, Alex Dranovsky, E. David Leonardo

**Affiliations:** Dranovsky-Leonardo (ADL) Laboratory, Department of Psychiatry, Division of Integrative Neuroscience, Columbia University and the New York State Psychiatric Institute, New York, NY (Drs Garcia-Garcia and Navarro-Sobrino, Ms Pilosof, Dr Dranovsky, and Dr Leonardo); Forest Research Institute, an Allergan affiliate, Jersey City, NJ (Dr Banerjee).

**Keywords:** vilazodone, 5-HT_1A_, fluoxetine, novelty suppressed feeding

## Abstract

**Background:**

Differences in 5-HT_1A_ receptor function have been implicated in vulnerability to depression and in response to treatment. Adding 5-HT_1A_ partial agonists to selective serotonin reuptake inhibitors has been touted as a strategy to increase their efficacy. Here we use the novelty suppressed feeding paradigm to compare the effects of vilazodone, a high-potency selective serotonin reuptake inhibitor, with high affinity for 5-HT_1A_ receptors to the reference selective serotonin reuptake inhibitor fluoxetine across several mouse strains that differ in their response to selective serotonin reuptake inhibitors.

**Methods:**

To confirm 5-HT_1A_ agonist activity, body temperature was measured after acute administration of vilazodone or fluoxetine, as administration of 5-HT_1A_ agonists induces hypothermia. We next used 3 strains of mice to examine the effects of the drugs on latency in the novelty suppressed feeding, a paradigm generally sensitive to chronic but not acute effects of antidepressants.

**Results:**

Vilazodone induces robust hypothermia and blocks stress-induced hyperthermia in a 5-HT_1A_-dependent manner, consistent with agonist effects at 5-HT_1A_ autoreceptors. In 129SvEv mice, vilazodone (10mg/kg/d) reduces the latency to eat in the novelty suppressed feeding test within 8 days, while no effect of fluoxetine (20mg/kg/d) was detected at that time. In contrast, both vilazodone and fluoxetine are effective at decreasing latency to eat in the novelty suppressed feeding paradigm in a strain with low autoreceptor levels. In mice with higher autoreceptor levels, no significant difference was detected between fluoxetine and vehicle (*P=.*8) or vilazodone and vehicle (*P*=.06).

**Conclusion:**

In mice, vilazodone may offer advantages in time of onset and efficacy over a reference selective serotonin reuptake inhibitor in the novelty suppressed feeding test.

## Introduction

Depression and anxiety are chronic and recurring illnesses that strike early in life and are thus amongst the leading causes of disability worldwide (Murray et al., 2012). While currently approved medications with antidepressant/anxiolytic activity are effective for some patients, many patients do not respond to initial treatment with a first-line medication. In addition, while most first-line treatments target some aspect of monoaminergic modulatory systems, a specific understanding of why individual drugs with antidepressant/anxiolytic activity are effective in some patients but not others remains an unsolved problem. Indeed, a physician’s first choice of medication is often influenced more by side effect than by efficacy profiles. As a result, there are often delays in obtaining optimal treatment, resulting in excess morbidity and suffering. The ability to select a medication both on considerations of likely efficacy and tolerability would be a great step forward. To this end, an improved understanding of the mechanistic differences between available medications is critical.

Here we compare the effects of vilazodone, a high efficacy serotonin reuptake inhibitor with potent 5-HT_1A_ partial agonist properties, to fluoxetine, the prototype selective serotonin reuptake inhibitor (SSRI) approved for the treatment of depression, in the novelty suppressed feeding (NSF) paradigm. This paradigm has previously been shown in mice to be sensitive to chronic but not acute treatment with several classes of antidepressants in a variety of mouse strains (Dulawa and Hen, 2005; Samuels and Hen, 2011). Several lines of evidence suggest that adding a 5-HT_1A_ partial agonist to SSRIs would augment the antidepressant effect (Celada et al., 2004), and there is some evidence to suggest that 5-HT_1A_ agonists alone may have antidepressant effect (Wilcox et al., 1996; Bielski et al., 2008). In addition, several genetics studies in humans suggest that variation in the gene that encodes the 5-HT_1A_ receptor may be important in determining both the vulnerability to depression and the response to treatment with antidepressants (Le Francois et al., 2008; Richardson-Jones et al., 2010). These studies are additionally supported by imaging studies in humans that suggest that 5-HT_1A_ receptor expression patterns may predict response to treatment with antidepressants (Miller et al., 2013). Indeed, previous work in mice has shown that mice with relatively higher levels of 5-HT_1A_ autoreceptors do not respond to fluoxetine in the NSF paradigm when compared with otherwise identical mice with lower levels of 5-HT_1A_ autoreceptors (Richardson-Jones et al., 2010). Here we hypothesized that vilazodone would be effective in the NSF paradigm under conditions that are resistant to treatment with fluoxetine.

## Methods

### Animal Husbandry

Animals were housed in groups of 3 to 5 per cage and had ad libitum access to food and water. Animals were maintained on a 12:12 light/dark schedule. Animal protocols were approved by the Institutional Animal Care and Use Committee at the New York State Psychiatric Institute and were conducted in accordance to the NIH Guide for the Care and Use of Laboratory Animals.

129SvEv/Tac mice were directly obtained from Taconic Inc. (Germantown, NY)

### Generation of the Conditional 5-HT_1A_ Autoreceptor Mice

To obtain a regulatable 5-HT_1A_ autoreceptor mouse, tetO-1A mice were bred to a transgenic mouse line with tTS expression driven from Pet promoter fragments as previously described (Richardson-Jones et al., 2010, 2011). tetO-1A+/+/Pet-tTS+ (1A-Hi) mice maintained in the presence of doxycycline (DOX) displayed no receptor suppression and with a pattern of expression that is indistinguishable from Pet-tTs- mice. Mice that have DOX withdrawn at P50 display an approximately 30% decrease in autoreceptor expression after 4 weeks (1A-Low) (Richardson-Jones et al., 2010, 2011).

### Drugs and Treatments

(±)-8-hydroxy-2-(di-n-propylamino) tetralin hydrobromide (8-OH-DPAT) and N-[2-[4-2-methoxyphenyl)-1-piperazinyl]ethyl]- N-2-pyridinil-cyclohexanecarboxamide maleate (WAY 100,635) were obtained from Sigma-Aldrich (St. Louis, MO). Vilazodone and citalopram were obtained from Forest Laboratories (Forest Research Institute, Jersey City, NJ) while fluoxetine hydrochloride was purchased from BIOTREND Chemicals AG (Wangen, Zurich). Both drugs were dissolved in water and administered via gavage. Fluoxetine 20mg/kg/d was chosen, as our experience suggests that doses between 18 and 20mg/kg/d routinely give the most robust responses in this paradigm. This dose range produces blood levels similar to those seen in patients treated with up to 80mg/d of fluoxetine, and higher doses exhibit nonlinear kinetics in mice (Dulawa et al., 2004).

### Behavioral and Physiological Testing

All animals used for behavioral testing were age matched within 2 weeks. Animals were males and initially tested at 14 to 16 weeks of age. For 8-OH-DPAT and stress-induced hyperthermia, drugs were given via i.p. injection. For the NSF test, drugs were given via gavage for up to 28 days. Testing in the NSF occurred on day 8 and 28 of treatment.

### 8-OH-DPAT–Induced Hypothermia

Mice were singly housed in clean cages for 10 minutes and 3 baseline body temperature measurements were taken. Ten minutes after the third baseline measurement, animals received different drugs i.p. Change in core temperature was assessed using a rectal probe every 10minutes for 60minutes as previously described (Richardson-Jones et al., 2010, 2011). 8-OH-DPAT is the prototypical 5-HT_1A_ full agonist. Although it does have some activity at 5-HT_7_ receptors (Landry et al., 2006), 8-OH-DPAT–induced hypothermia in mice is dependent on functional 5-HT_1A_ autoreceptors (Richardson-Jones et al., 2011).

### Stress-Induced Hyperthermia

Stress-induced hyperthermia was performed as previously described (Richardson-Jones et al., 2010). Briefly, animals in their home cages were moved to a testing room and allowed to acclimate for 1 hour. One animal per cage was removed, and a baseline body temperature was measured rectally immediately prior to administration of the appropriate drug. Each animal was then placed in a novel, clean cage for 10 minutes, after which a second body temperature was recorded.

### NSF

The NSF paradigm is a test of hyponeophagia that measures the latency of a mouse to consume food placed in the middle of a brightly lit, aversive arena (Bodnoff et al., 1988). Testing was performed as previously described (David et al., 2009; Richardson-Jones et al., 2010). Briefly, animals were food restricted for 24 hours, and the latency (dependent measure) to begin chewing a food pellet placed on a white piece of filter paper (12.5-cm diameter) in the center of brightly lit arena was recorded (40-×60-cm arena with 2cm of new corn cob bedding; 800–900 lux). The trial was terminated either when an animal began chewing or 300 seconds transpired. Immediately after terminating the trial, animals were placed in their home cage, and the amount of food consumed in 5 minutes was measured (home cage consumption), followed by an assessment of postrestriction weight. Percentage body weight lost during food deprivation prior to the testing was assessed to ensure both groups lost similar amounts of weight. Home cage consumption immediately after testing was assessed as a relative measure of hunger.

### Statistical Analysis

Results from data analyses were expressed as mean ± SEM. One-tailed *P* < .05 was used as the threshold for significance. Two-way ANOVA with repeated measures in 1 factor was used for 8-OH-DPAT–induced hypothermia where drugs were expected to decrease temperatures more than vehicle (Richardson-Jones et al., 2011). ANOVAs were followed up with Fisher’s protected least squares difference posthoc comparisons. For NSF, the hypothesis was that drugs would decrease the latency to feed (Richardson-Jones et al., 2010). Data in several groups were right censored and thus analyzed using a Kaplan-Meier survival analysis with the Gehan-Breslow-Wilcoxon method. Bonferroni corrected *P* values are presented.

## Results

### Vilazodone Acts as an Agonist at 5-HT_1A_ Autoreceptors in Mice

**Figure 1.**
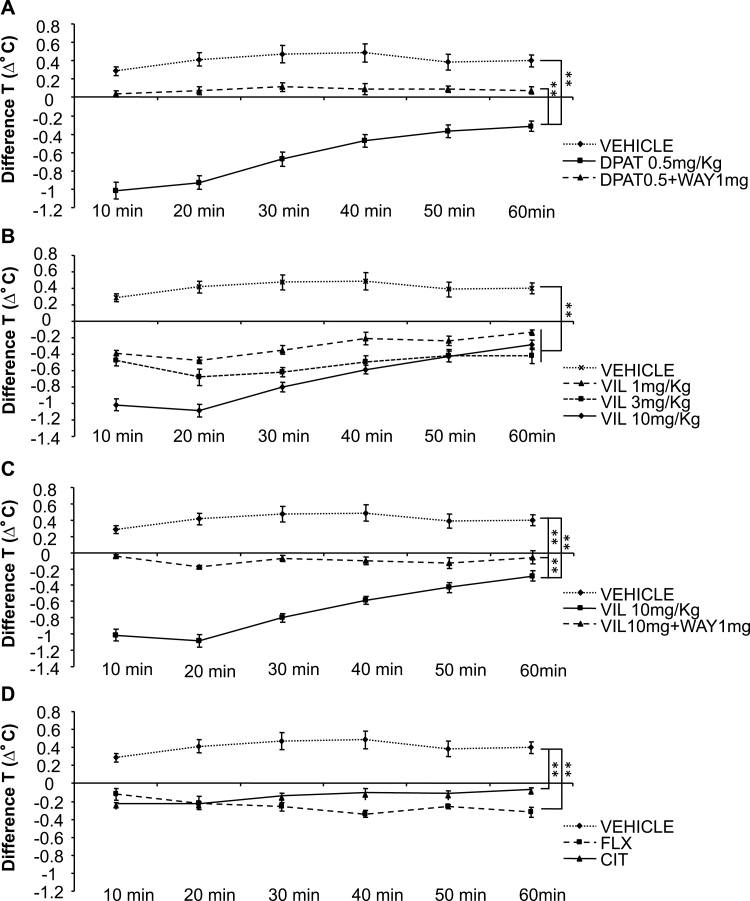
5-HT_1A_ receptor agonist-induced hypothermia in mice: reversal by 5-HT_1A_ receptor antagonists. (A) The 5-HT_1A_ receptor agonist 8-OH-DPAT produced a hypothermic response in mice, and this effect was significantly reversed/antagonized by the 5-HT_1A_ receptor antagonist WAY 100635 (n = 7–8 mice/group). (B) Mice injected with vilazodone [VIL] 1, 3, and 10mg/kg showed dose-dependent decreases in body temperature (n = 5–8 mice/group). (C) The hypothermic response to vilazodone was significantly attenuated by co-administration with the 5-HT_1A_ receptor antagonist WAY 100635, indicating that the hypothermic response to vilazodone is due its effects at 5-HT_1A_ receptors (n = 7 mice/group). (D) Effect of conventional selective serotonin reuptake inhibitors (SSRIs) (fluoxetine [FLX] and citalopram [CIT]) (n = 5–8 mice/group; *P<.05, **P<.01).

5-HT_1A_ agonists induce a rapid hypothermic response when injected systemically. In mice this effect is due to activation of autoreceptors (Richardson-Jones et al., 2011; Ferres-Coy et al., 2013). To test the effect of vilazodone at 5-HT_1A_ autoreceptors, we evaluated its ability to induce hypothermia in the presence or absence of WAY 100,635 (5-HT_1A_ antagonist), compared to both a reference 5-HT_1A_ agonist 8-OH-DPAT, and to reference SSRIs, fluoxetine, and citalopram (ANOVA main effect of treatment: F_8,53_=46.928; *P*<.01, main effect of time: F_5,53_=57.724; *P<.*01: treatment x time interaction: F_40,53_=13.506; *P<.*01). As expected, 8-OH-DPAT produced a robust hypothermic response (Fisher posthoc: *P<.*01 8-OH-DPAT vs vehicle) that was reversed by the 5-HT_1A_ receptor antagonist WAY 100,635 (Fisher posthoc: *P<.*01 8-OH DPAT+WAY vs 8-OH DPAT alone; n = 7–8 mice/group) (Figure 1A). Vilazodone showed a dose-dependent difference from vehicle at 1, 3, and 10mg/kg (Fisher posthoc: *P<.*01 all doses vs vehicle; n = 5–8 mice/group) (Figure 1B). This difference included a significant hypothermic response (maximal response seen at 20 minutes, with 0.4 [1.86%], 0.6 [2.52%], and 1.2degrees [3.73%] below the original baseline temperature, respectively). Furthermore, the hypothermic response of vilazodone 10mg/kg was significantly attenuated by co-administration with WAY 100,635 (Fisher posthoc: *P<.*01 Vil10 vs Vil10+WAY), although the effect was not abolished (Fisher posthoc *P<.*01 vehicle vs Vil10+WAY; n = 7 mice/group) (Figure 1C). These results suggest that the hypothermic response of vilazodone was largely due to its agonist effects at 5-HT_1A_ receptors. At high doses, the conventional SSRIs, fluoxetine and citalopram, also induce hypothermia (Fisher posthoc: *P<.*01 SSRIs vs vehicle; n = 5–8 mice/group) (Figure 1D). However, this effect is significantly less than the effect seen with vilazodone at 10mg/kg (Fisher posthoc: *P<.*01 vilazodone 10mg/kg vs SSRIs) (supplementary Figure 1A).

### Vilazodone Attenuates Stress-Induced Hyperthermia in a 5-HT_1A_ Receptor-Dependent Fashion

**Figure 2.**
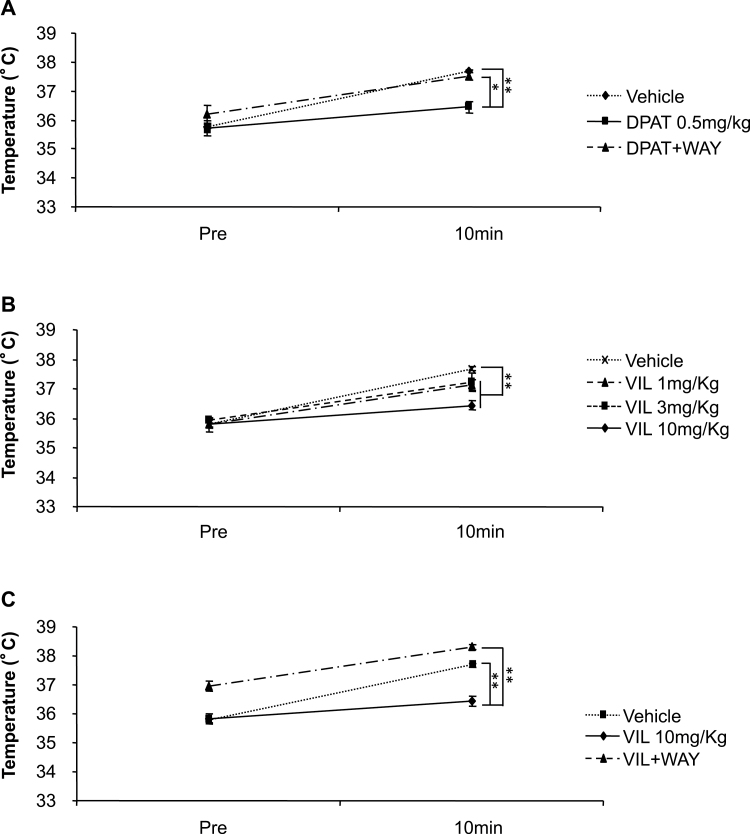
Attenuation of stress-induced hyperthermia by 5-HT_1A_ receptor agonists in mice. (A) Stress-induced hyperthermia was attenuated by the 5-HT_1A_ agonist 8-OH-DPAT; this effect of 8-OH-DPAT was antagonized by WAY 100,635 (n = 4–5 mice/group). (B) Vilazodone 1, 3, and 10mg/kg significantly attenuated stress-induced hyperthermia to a novel cage (n = 5 mice/group). (C) Co-administration with WAY 100,635 diminishes the effects of vilazodone (n = 5 mice/group). Mean hyperthermic response to vehicle=1.9±06^o^C, vil 10mg/kg = .62±04^o^C, and vil+WAY = 1.33±.11^o^C. (*P<.05; **P<.01).

The stress-induced hyperthermia paradigm has been validated as an anxiety model that is responsive to treatment with 5-HT_1A_ agonist and benzodiazepine drugs but not responsive to acute treatment with SSRI drugs (Vinkers et al., 2008). Accordingly, stress-induced hyperthermia was attenuated by 8-OH-DPAT to 60.4% of vehicle levels. (ANOVA for main effect of treatment: F_2,11_=18.319; *P<.*01; Fisher posthoc: *P<.*01 8-OH-DPAT vs vehicle), and this effect was antagonized by WAY 100,635 to 32.4% of the vehicle group (Fisher posthoc: *P<.*05 8-OH-DPAT vs 8-OH-DPAT+WAY; n = 4–5 mice/group) (Figure 2A). Vilazodone was able to significantly attenuate stress-induced hyperthermia to a novel cage (1mg/kg: 28.4%; 3mg/kg: 31.6%; 10mg/kg: 67.4% of vehicle levels; ANOVA for main effect of treatment: F_3,16_=13.765; *P<.*01 all doses vs vehicle; n = 5 mice/group) (Figure 2B). The decrease in the hyperthermic response induced by vilazodone at 10mg/kg was attenuated by co-administration of WAY 100,635 to 29.9% of vehicle (ANOVA for main effect of treatment: F_2,2_=72.485; *P<.*01; Fisher posthoc: *P<.*01 for all groups; n = 5 mice/group) (Figure 2C). These results demonstrate that vilazodone attenuates stress-induced hyperthermia through a 5-HT_1A_ receptor-dependent mechanism.

### Vilazodone Elicits an Accelerated Effect in the NSF Paradigm Compared with Fluoxetine

**Figure 3.**
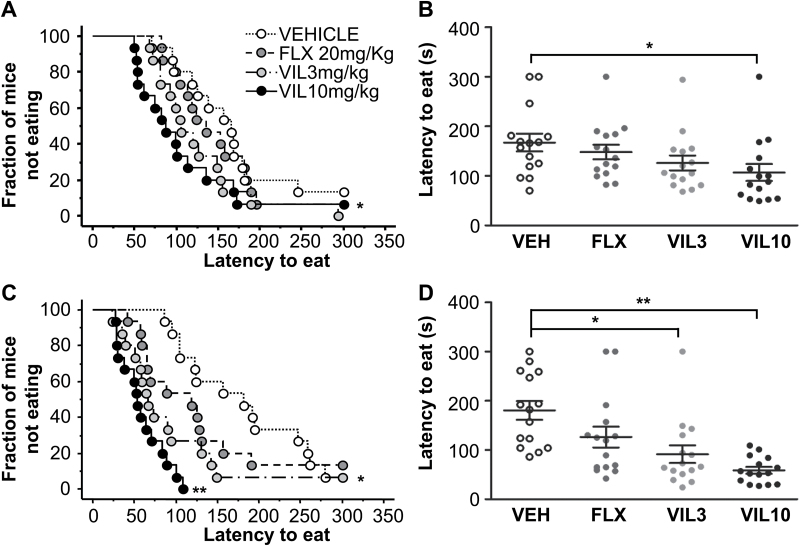
129SvEv Mice: subchronic and chronic treatment effect on feeding latency in novelty suppressed feeding (NSF). (A-B) Following a subchronic, 8-day treatment, a response was observed in mice treated with vilazodone 10mg/kg as evidenced by lower latency to feed relative to vehicle-treated controls in the NSF test. Fluoxetine did not significantly affect latency to feed in this test (n = 15 mice/group). (C-D) A robust response was also observed in mice following chronic, 28-day treatments with vilazodone 3 and 10mg/kg relative to vehicle-treated controls in the NSF test. The difference in latency elicited by chronic fluoxetine administration at 20mg/kg was not significant (n = 15 mice/group). (A-D) Kaplan-Meier cumulative survival curve. (B-D) Scatter graph depicting individual animal latencies with mean and SEM (*P<.05 vs vehicle; **P<.01 vs vehicle).

The NSF paradigm has 2 features that have made it useful in modeling the response to antidepressants: (1) It is generally sensitive to chronic (>3 weeks) but not acute administration of drugs with antidepressant efficacy (Wang et al., 2008), and (2) the response is affected by the genetic background of the mice (Lucki et al., 2001), with some strains not responding to SSRIs in this paradigm (Ibarguen-Vargas et al., 2008). Here, using the 129SvEv inbred mouse strain, we tested the effect of several doses of vilazodone. After 8 days, no effect of treatment was detected for fluoxetine at 20mg/kg (Gehan-Breslow-Wilcoxon Bonferroni corrected *P=.*816). The effect of vilazodone was not significant at 3mg/kg (Gehan-Breslow-Wilcoxon Bonferroni corrected *P=.*069) but was significant at 10mg/kg (Gehan-Breslow-Wilcoxon Bonferroni corrected *P=.*01; n = 15 mice/group) (Figure 3A-B). After 28 days of treatment, the expected effect of 20mg/kg fluoxetine was not significant (Gehan-Breslow-Wilcoxon Bonferroni corrected *P=.*079), while vilazodone elicited a significant effect relative to vehicle at both 3mg/kg and 10mg/kg (Gehan-Breslow-Wilcoxon Bonferroni corrected *P<.*001 for both; n = 15 mice/group) (Figure 3C-D). The effect on latency cannot be attributed to changes in hunger between groups, as we did not detect any change in home cage consumption after the 8- or 28-day tests (supplementary Figure 2A-B). Thus, in the 129SvEv strain, vilazodone elicits a faster and more robust response than fluoxetine in the NSF paradigm.

### Effect of 5-HT_1A_ Autoreceptor Levels on Response to Vilazodone in the NSF Paradigm

**Figure 4.**
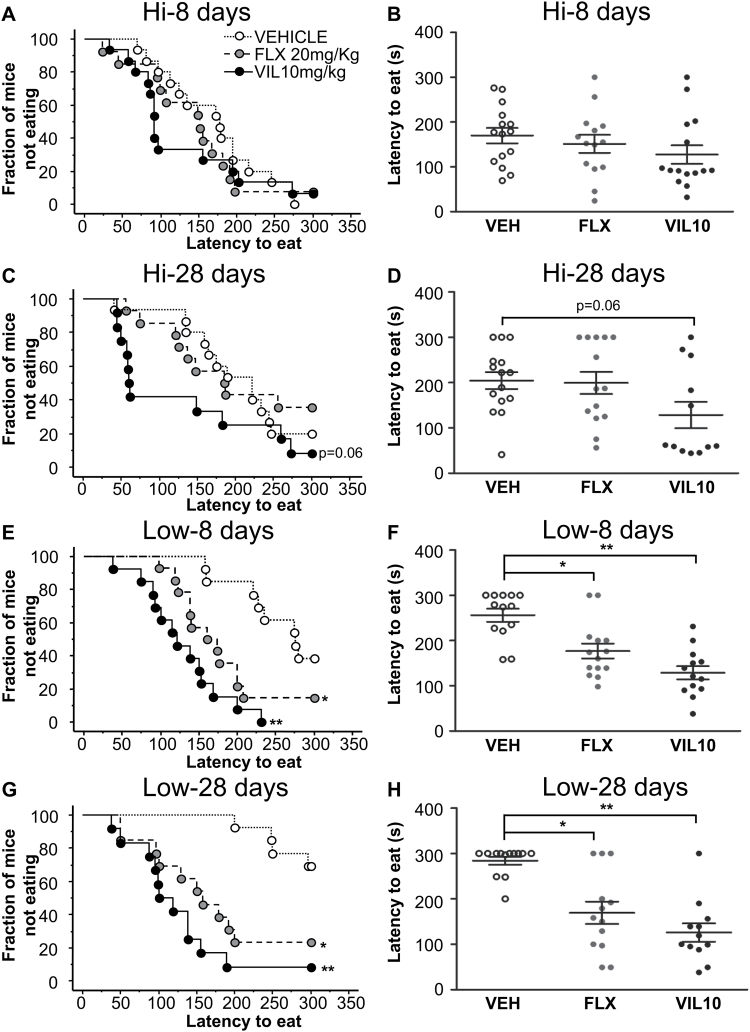
tetO-1A+/+/Pet-tTS+ mice on DOX continuously (Hi) and off DOX at P50 (Low): subchronic and chronic treatment effect on feeding latency in novelty suppressed feeding. At 8 days, no difference in mean latency was detected between vehicle and either fluoxetine (20mg/kg) or vilazodone (10mg/kg) in 5-HT_1A_ Hi mice. (A-B) At 28 days of treatment, vehicle and fluoxetine groups have similar latencies. The vilazodone group has a lower latency that is not statistically different from the vehicle group (C,D) (8 days: n = 14–15 mice/group; 28 days: n = 12–15 mice/group). (E-F) The 5-HT_1A_ Low mice responded robustly to both fluoxetine (20mg/kg) and vilazodone (10mg/kg) after only 8 days of treatment (n = 13–14 mice/group). (G-H) In 5-HT_1A_ Low mice, chronic (28-d) treatment with either fluoxetine or vilazodone significantly reduced feeding latency in the novelty suppressed feeding (NSF) arena (n = 12–13 mice/group). (A,C,E,G) Kaplan-Meier cumulative survival curve. (B,D,F,H) Scatter graph depicting individual animal latencies with mean and SEM (*P<.05 vs vehicle, **P<.01 vs vehicle).

We next examined the effects of vilazodone in 2 strains of mice that are identical except for their 5-HT_1A_ autoreceptor expression profile. The strains are engineered to allow for DOX-dependent temporal control of 5-HT_1A_ autoreceptor expression (Richardson-Jones et al., 2010). In the presence of DOX, (5-HT_1A_ Hi) 5-HT_1A_ autoreceptor levels in the engineered animals are indistinguishable from their nontransgenic littermates. In the absence of DOX, 5-HT_1A_ expression levels are decreased by about 30% (5-HT_1A_ Low). We have previously demonstrated that 5-HT_1A_ Hi mice do not respond to fluoxetine in the NSF paradigm. Here, no effect of treatment is observed at 8 days for either fluoxetine or vilazodone (Gehan-Breslow-Wilcoxon Bonferroni corrected *P=.*64 and *P=.*15, respectively; n = 14–15 mice/group) (Figure 4A-B). At 28 days, no effect of fluoxetine was detected (Gehan-Breslow-Wilcoxon Bonferroni corrected *P=.*88). The vilazodone effect at 10mg/kg missed the cutoff for significance (Gehan-Breslow-Wilcoxon Bonferroni corrected *P=.*06; n = 12–15 mice/group) (Figure 4C-D). Home cage consumption was decreased in the fluoxetine group after 8 days, but not in the other groups (supplemental Figure 3A-B).

Finally, we examined the effects on the NSF by treating the 5-HT_1A_ Low mice with the same drugs. In this strain, both fluoxetine 20mg/kg and vilazodone 10mg/kg were superior to saline after only 8 days of treatment (Gehan-Breslow-Wilcoxon Bonferroni corrected: *P=.*003 and *P<.*0001, respectively; n = 13–14 mice/group) (Figure 4E-F). At 28 days, both fluoxetine (20mg/kg) and vilazodone (10mg/kg) showed a robust response (Gehan-Breslow-Wilcoxon Bonferroni corrected: *P=.*0009 and *P<.*0001; n = 12–13 mice/group) (Figure 4G-H). Finally, no differences were detected in home cage consumption after the 8- or 28-day tests (supplemental Figure 3C-D).

## Discussion

The efficacy of vilazodone as an antidepressant is hypothesized to be a result of its combined ability to block the reuptake of serotonin and its ability to act as a partial agonist at 5-HT_1A_ receptors. Here we use a 5-HT_1A_ agonist-induced hypothermia paradigm to demonstrate that vilazodone has significant in vivo effects at 5-HT_1A_ autoreceptors above and beyond those that would be expected from increased serotonin due to 5-HT transporter blockade. In addition, we demonstrate the ability of vilazodone to block stress-induced hyperthermia in a 5-HT_1A_-dependent manner. Our behavioral data demonstrate that chronic treatment with vilazodone at doses that exhibit significant 5-HT_1A_ agonism results in decreased latencies to eat in the NSF paradigm that differ in both timing and effect size to the response to fluoxetine in 3 selected mouse strains. Consistent with previous studies, we saw 3 distinct patterns of response in the NSF to fluoxetine in the strains tested. The 5-HT_1A_ Low mice were highly responsive to fluoxetine with effects seen both subchronically at 8 days and chronically at 28 days. The 129SvEv strain was moderately responsive with no effect seen subchronically and an effect usually emerging by 4 weeks. In this particular cohort, this effect narrowly missed the cutoff for significance. Finally, the 5-HT_1A_ Hi strain was completely unresponsive in this paradigm (Santarelli et al., 2003; Richardson-Jones et al., 2010). Interestingly, in the case of vilazodone, both the 129SvEv and the 5-HT_1A_ Low mice were highly responsive, with the 10mg/kg of vilazodone eliciting an effect as early as 8 days into treatment. The 5-HT_1A_ Hi mice were least affected by treatment with vilazodone, with decreased mean latencies that were not statistically distinct from the vehicle group. Interestingly, in the 5-HT_1A_ Hi mice, the distribution of latencies in the NSF in response to vilazodone at both 8 days and at 28 days appeared bimodal. About one-half of the animals exhibited a very low latency, raising the possibility that a subset of animals in this group may be responding. Such a bimodal distribution in the NSF, with responders and nonresponders within the same strain has previously been described. Finally, the absolute mean latencies of the vilazodone-treated animals were lower than the respective means of both the vehicle and fluoxetine-treated animals in all cohorts. Taken together, these data suggest that vilazodone has a more robust and potentially earlier effect in the NSF across multiple strains compared with fluoxetine.

One explanation that has been given for the delayed response to treatment with SSRI drugs in depression and in some behavioral assays is the need to desensitize 5-HT_1A_ autoreceptors before a treatment effect can be observed (Blier et al., 1998). The accelerated effect of vilazodone relative to fluoxetine in the NSF in the 129SvEv strain might be interpreted in the context of the results with the 5-HT_1A_ Low mice, where fluoxetine also acts in an accelerated manner. We previously demonstrated that as a result of low autoreceptor levels, the 5-HT_1A_ Low mice behave functionally as if they have desensitized autoreceptors (Richardson-Jones et al., 2010), providing a rationale for an early response to SSRIs in this strain. In the case of the 129SvEv strain, it is possible that vilazodone but not fluoxetine is able to generate a subacute response at 8 days, because vilazodone has been shown to promote rapid desensitization of 5-HT_1A_ receptors (Ashby et al., 2013). A rationale for the relative resistance of the 5-HT_1A_ Hi mice is less clear, although the potential bimodal distribution of latencies in vilazodone treated mice merits further investigation.

In general, it has been difficult to predict when one treatment for depression might work over another treatment in any given patient. Indeed, this lack of a personalized approach has generally led to the prescription of antidepressants using other criteria such as cost or side effect profiles. Together with data from imaging and genetic studies in humans, our data in the NSF model in mice support a hypothesis in which vilazodone and SSRIs may have equivalent effects in populations with lower levels of 5-HT_1A_ autoreceptors, while vilazodone may have an advantage in patients that do not respond to SSRIs. Furthermore, our data are consistent with the hypothesis that higher levels of autoreceptors increase resistance to SSRIs (Le Francois et al., 2008).

## Statement of Interest

E.D.L. previously provided consulting services for PGx Health. P.B. was an employee of Forest Research Institute at the time of funding. There are no other conflicts to disclose.

## Supplementary Material

supplementary Figure 1A
