# Supplementary material for: 5-HT1A Agonist Properties Contribute to a Robust Response to Vilazodone in the Novelty Suppressed Feeding Paradigm
Source: Int J Neuropsychopharmacol. 2016 Jun 28;19(10):pyw057. doi: 10.1093/ijnp/pyw057 (PMC5091830; doi:10.1093/ijnp/pyw057)
Supplement: supplementary Figure 1A [file FINAL_resub_SUPPLEMENTAL_VIL_PAPER_April16.docx]

**Supplemental Material**

**5-HT_1A_ agonist properties contribute to a robust response to vilazodone in the novelty suppressed feeding paradigm**

**Alvaro L. Garcia-Garcia, Miriam Navarro-Sobrino, Gila Pilosof, Pradeep Banerjee, Alex Dranovsky, E. David Leonardo.**


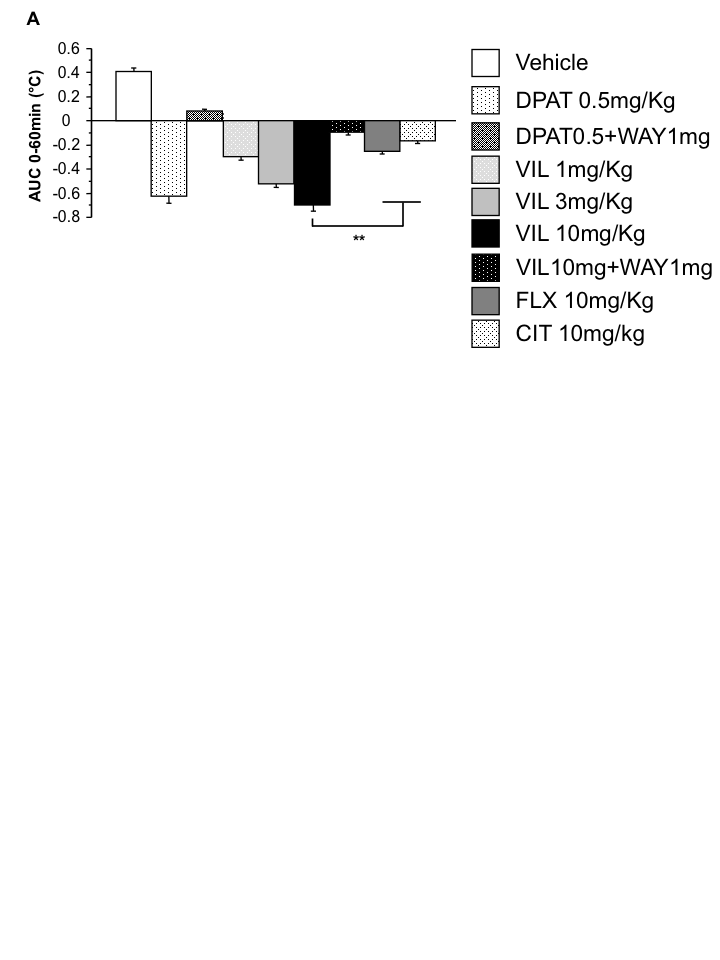
**Supplemental Figure S1.**

**Average change in body temperature after drug administration.**

Following an acute injection of drug at T_0_ core body temperature was measured every 10 minutes for an hour. The mean change in temperature is plotted above for each injection. The significant difference between Vil 10mg/kg and fluoxetine 10mg/kg and Citalopram 10mg/kg is highlighted. **p<0.01. Within group comparisons are shown in Figure 1.


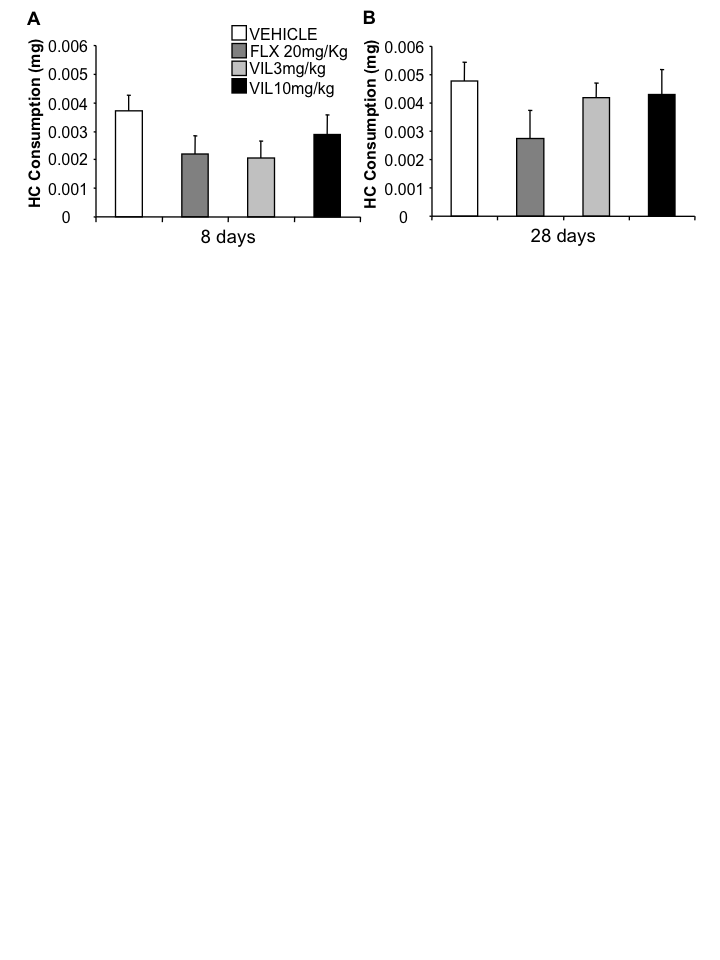


**Supplemental Figure S2. Sub-chronic and chronic treatment effect on Home Cage Consumption in Novelty Suppressed Feeding.** **(A,B)** Following a sub-chronic or chronic treatment (8-day or 28-day, respectively), no difference was observed between groups in home cage consumption. (ANOVA for main effect of treatment 8 days: F_3,56_=1.428; p=0.2443; 28 days: F_3,56_=1.193; p=0.3207) (*n* = 15 mice per group). Bar graphs represent mean and S.E.M.


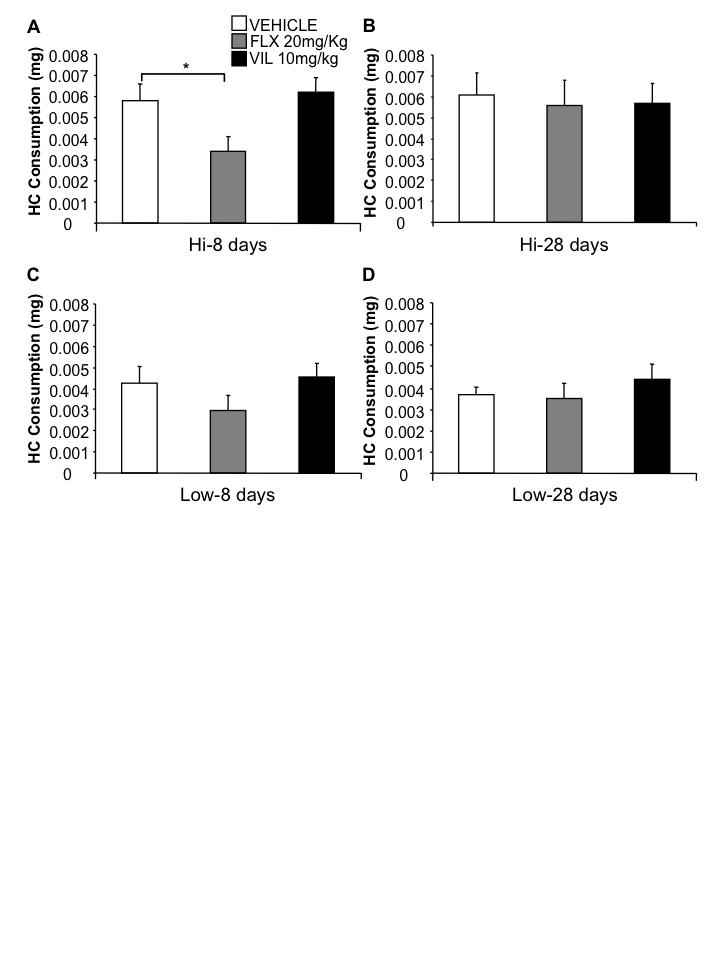


**Supplemental Figure S3. Sub-chronic and chronic treatment effect on home cage in the novelty suppressed feeding test in 5-HT_1A_ Hi and Low mice. (A)** Following a sub-chronic treatment (8-day), fluoxetine decreases home cage consumption after the novelty suppressed feeding test in 5-HT_1A_ Hi mice (ANOVA for main effect of treatment: F_2,41_=3.887; p<0.05; Fisher post hoc: p<0.05 Fluoxetine vs Vehicle) (*n* = 14-15 mice per group). **(B)** No difference was observed in home cage consumption in 5-HT_1A_ Hi mice after chronic treatment (ANOVA for main effect of treatment: F_2,38_=0.068; p=0.9347). (*n* = 12-15 mice per group). **(C,D)** Following a sub-chronic and chronic treatment (8-day or 28-day, respectively), no difference was observed in home cage consumption in 5-HT_1A_ Low mice. (ANOVA for main effect of treatment 8-day: F_2,37_=1.581; p=0.2194; 28-day: F_2,35_=0.656; p=0.5251) (8 day: *n* = 13-14 mice per group; 28 day: *n* = 12-13 mice per group). Bar graphs represent mean and S.E.M. (*p<0.05 vs. vehicle; **p<0.01 vs. vehicle).
